# Regulation of long non-coding RNAs by essential oils: Therapeutic implications in human diseases

**DOI:** 10.1590/1678-4685-GMB-2025-0181

**Published:** 2026-04-03

**Authors:** Lívia da Cunha Agostini, Mariane Ster da Silva Teixeira, Glenda Nicioli da Silva

**Affiliations:** 1 Universidade Federal de Ouro Preto, Escola de Farmácia, Programa de Pós-Graduação em Ciências Farmacêuticas (CiPharma), Ouro Preto, MG, Brazil.; 2 Universidade Federal de Ouro Preto, Escola de Farmácia, Departamento de Análises Clínicas (DEACL), Ouro Preto, MG, Brazil.

**Keywords:** Essential oils, human diseases, long non-coding RNA, natural therapy

## Abstract

Essential oils are natural substances with broad therapeutic potential due to their antioxidant, anti-inflammatory, and anticancer properties. Recent studies have demonstrated that these compounds can modulate the expression of non-coding RNAs, such as lncRNAs, miRNAs, and circRNAs, which play crucial roles in post-transcriptional and epigenetic regulation in various pathological conditions. This modulation influences key biological processes, including apoptosis, drug resistance, inflammation, cell differentiation, and cognitive function. Therefore, the regulation of ncRNAs by essential oils emerges as a promising strategy for the development of novel therapeutic approaches in cancer, respiratory, joint, and neurodegenerative diseases. This review summarizes the latest findings on the ability of essential oils to modulate ncRNAs, highlighting their potential in the prevention and treatment of a wide range of human diseases.

## Essential oils

According to the European Pharmacopoeia, essential oils are defined as aromatic products of complex chemical composition, obtained from plant raw materials through processes such as distillation and pressing. These oils have a characteristic aroma of the plant of origin and are mainly composed of a combination of terpenes, alcohols, esters, aldehydes, ketones, and phenols. In the 16th century, Paracelsus (1493-1541) referred to essential oils as the “soul of the plant” or “quintessence for healing”, believing that distillation extracted the plant’s healing essence (de Sousa et al., 2023). 

Essential oils are composed of a wide variety of compounds, potentially containing more than 300 distinct components derived from the plant’s secondary metabolism. The concentrations of these constituents vary depending on genetic, physiological, and environmental factors, including the plant’s developmental stage, climate, soil conditions, and extraction method. Although the major compounds are often associated with the primary biological activities, minor constituents also play significant roles through synergistic interactions (Dhifi et al., 2016). 

The constituents of essential oils have different metabolic precursors and are synthesized via distinct biosynthetic pathways. They can be terpenoids, derived from the mevalonic acid and methylerythritol phosphate pathways, such as sesquiterpenes, or non-terpenoids (Jaeger and Cuny, 2016; de Sousa et al., 2023). For example, turmeric essential oil has been used in the treatment of liver fibrosis and cancer, showing effects in reducing oxidative stress and improving mitochondrial dysfunction in depressed rats (Zeng et al., 2020; Chen et al., 2021; Lai et al., 2024). Similarly, *Brucea javanica* oil has been associated with promising outcomes in gastric cancer treatment, including improved overall response rates, clinical benefit, and reduced adverse drug reactions, as well as promoting autophagy in ovarian cancer cells (Wang et al., 2021b; Wang et al., 2022). β-Patchoulene essential oil has demonstrated anti-inflammatory activity and modulated metabolic disease-related markers in rats (Chen et al., 2017; Hong et al., 2020), while *Atractylodes lancea* exhibits anti-inflammatory and antioxidant properties (Li et al., 2023). 

Other relevant chemical groups include esters, such as linalyl acetate and geranyl acetate, as found in *Mentha suaveolens × piperita* (grapefruit mint), widely used in the pharmaceutical and food industries and associated with anticancer, antioxidant, and antifungal activities (Jahanafrooz et al., 2024). Among the phenols (e.g., thymol, carvacrol, and eugenol), *Thymus vulgaris* essential oil is notable for its antimicrobial, anti-biofilm, and wound-healing properties (Sakkas and Papadopoulou, 2017; Alsakhawy et al., 2022; Bakó et al., 2023), while *Monarda didyma L.* essential oil is recognized for its antioxidant and anti-inflammatory activities (Fraternale et al., 2022). Among the furanocoumarins, *Angelica sinensis* essential oil has shown promising results in improving cognitive function in mice with cerebral ischemia and in exerting anti-inflammatory effects (Li et al., 2022; J. Zhao et al., 2024). Lastly, aldehydes are also commonly found in essential oils, for instance, *Cinnamomum zeylanicum* oil exhibits antioxidant, antimicrobial, and anticancer properties (Saleem et al., 2015; Denkova-Kostova et al., 2021; Cappelli et al., 2023). 

The extraction method is crucial for preserving the properties of volatile compounds, and different techniques are employed depending on the nature of the plant material. Enfleurage is used for delicate flowers, where petals are placed on odorless fat to absorb volatile compounds, later extracted with alcohol. In hydrodistillation, the plant material is immersed in boiling water, and the steam carries the volatiles, which are then condensed and separated. Steam distillation involves passing water vapor through a chamber containing the plant. Organic solvent extraction uses non-polar solvents to dissolve aromatic compounds, which are isolated after solvent evaporation. Supercritical fluid extraction employs supercritical carbon dioxide as a selective solvent. Lastly, cold pressing, typically used for citrus fruits, involves the mechanical extraction of oils from the peels (López-Hortas et al., 2022). 

A single plant sample can be subjected to different extraction methods, yielding distinct chemical profiles. The analysis of essential oil constituents is often performed using gas chromatography coupled with mass spectrometry (GC-MS), which allows precise identification and quantification based on spectral profiles and comparison with plant reference standards (Thamer and Thamer, 2023). 

Due to their complex chemical composition, essential oils exhibit a range of pharmacological activities, including antioxidant, antiviral, antimicrobial, anti-inflammatory, anxiolytic, antidepressant, and antitumor effects (López-Hortas et al., 2022; de Sousa et al., 2023). These properties make them attractive candidates in the development of novel therapeutic agents, particularly in the context of rising antimicrobial resistance and the global search for natural treatment alternatives.

Essential oils offer a versatile natural therapy option, with various administration routes suited to different therapeutic needs. Topical application is indicated for skin conditions and localized pain relief; inhalation is effective for emotional and psychological benefits; oral administration is also possible, though it requires precise dosing to avoid toxicity (Caballero-Gallardo et al., 2025). 

The incorporation of essential oils into nanoparticle systems has emerged as an innovative strategy, enabling controlled release, enhanced bioavailability, and application via otherwise unfeasible routes due to the physicochemical properties of the compounds. Advanced systems, such as dendrimers, nanogels, nanosuspensions, and nanotubes, facilitate absorption via endocytosis and transcytosis (Soares et al., 2021). 

Brazil stands out as one of the most biodiverse countries in the world. Its biomes, the Amazon, Atlantic Forest, Cerrado, Caatinga, Pantanal, and Pampa, offer a vast array of aromatic and medicinal plant species with the potential to produce essential oils of high therapeutic value (Ferreira et al., 2022). 

In summary, essential oils represent a complex arsenal of bioactive compounds that act synergistically on multiple biological targets, conferring relevant therapeutic properties across a range of health conditions (Zhao et al., 2022; Zonfrillo et al., 2022). The growing interest in these natural compounds is driven not only by their pharmacological potential but also by their ability to serve as adjuvants or alternatives in diverse therapeutic contexts, underscoring the need for systematic studies to better understand their mechanisms of action, toxicity, and safe clinical applications (Abdullahi et al., 2020; Čmiková et al., 2023). 

## Structural features and role of non-coding RNAs

The central dogma of molecular biology, proposed by Francis Crick in 1958, describes the unidirectional flow of genetic information in which DNA is transcribed into RNA, and RNA is subsequently translated into proteins (Crick, 1958). However, with the advent of next-generation sequencing technologies, it was discovered that pervasive transcription occurs throughout the genome. Approximately 80% of the DNA is transcribed into RNA, yet only about 1.5% of these transcripts are translated into proteins (Hangauer, Vaughn and McManus 2013). 

**Figure 1:**
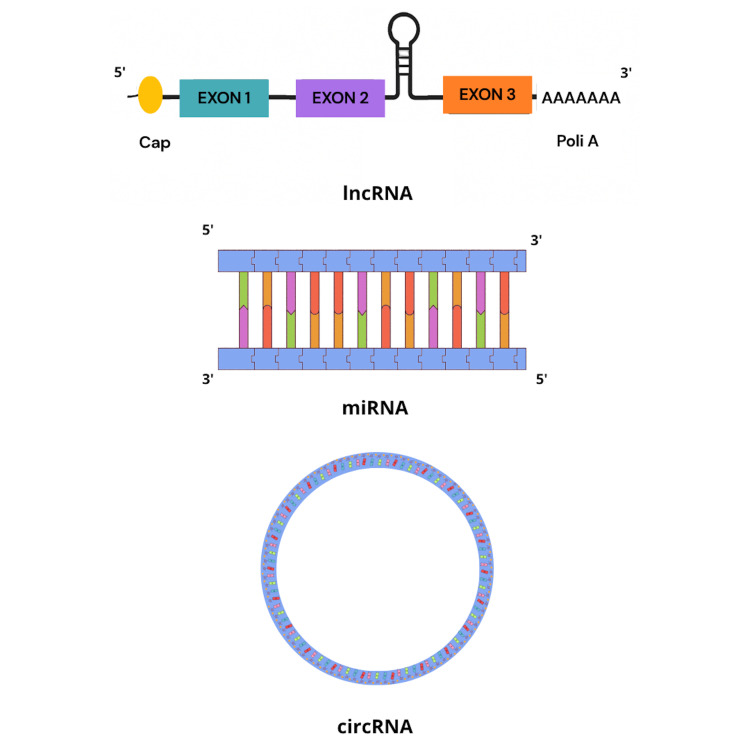
Structural representation of lncRNAs, miRNAs and circRNAs. Overview schematic of the structural features of key non-coding RNAs: long non-coding RNA (lncRNA), microRNA (miRNA), and circular RNA (circRNA).

RNAs are now broadly classified into two categories: coding RNAs, which have the potential to be translated into proteins, and non-coding RNAs (ncRNAs), which are not translated but are functionally active in the regulation of gene expression. ncRNAs are further subdivided based on transcript length into two main groups: those shorter than 200 nucleotides and those longer than 200 nucleotides (Loganathan and Doss, 2023). Among the most studied and functionally relevant classes of ncRNAs are long non-coding RNAs (lncRNAs), microRNAs (miRNAs), and circular RNAs (circRNAs), all of which play key roles in a wide range of physiological and pathological processes (Loganathan and Doss, 2023) (Figure 1).

**Figure 2:**
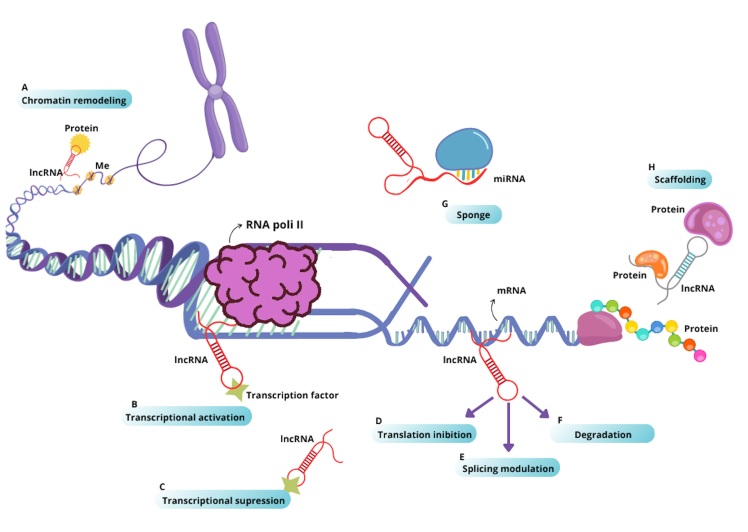
Main biological roles and regulatory functions of lncRNAs. Functional classification of lncRNAs. (A) They recruit protein components of chromatin remodeling complexes, modifying their organization; (B) They promote transcription by guiding transcription factors to promoters; (C) They can inhibit transcription by sequestering transcription factors. Furthermore, they interact with mRNAs; (D) Blocking their translation; (E) Modulating splicing; (F) Targeting them for degradation; (G) They act as “sponges” by binding to complementary miRNAs, neutralizing their effects; (H) They serve as scaffolds, providing anchoring points for proteins that act in concert.

Long non-coding RNAs (lncRNAs) are RNA molecules longer than 500 nucleotides and are classified based on their genomic location relative to protein-coding genes. They can be intergenic, antisense, or intronic (Mattick et al., 2023). LncRNAs perform a wide array of regulatory functions, including modulating histone modifications and DNA methylation in epigenetic processes; regulating transcriptional programs by directly interacting with the transcriptional machinery to activate or repress gene expression; inhibiting translation; modulating splicing; inducing mRNA degradation; acting as molecular sponges for miRNAs, thereby neutralizing their effects; and providing structural scaffolding for protein complexes (Salehi et al., 2017; Ritter et al., 2019; Liu et al., 2025) (Figure 2).

MicroRNAs (miRNAs) are small non-coding RNAs, typically 17 to 25 nucleotides in length, that function in the post-transcriptional regulation of gene expression, primarily by promoting the degradation or translational repression of target messenger RNAs (mRNAs) (Annese et al., 2020). MiRNAs are transcribed from DNA sequences located within exons, introns, or single host genes. After transcription, they form primary microRNAs (pri-miRNAs), which fold into hairpin structures and are recognized by the microprocessor complex, a protein complex composed of the RNase Drosha and DiGeorge Syndrome Critical Region 8 (DGCR8). This complex cleaves the pri-miRNA to produce a precursor miRNA (pre-miRNA) of approximately 60-100 nucleotides (Ota et al., 2013). 

The pre-miRNA is then exported to the cytoplasm via Exportin 5, where it is further processed by the enzyme Dicer, which cleaves it into a mature double-stranded miRNA molecule. One strand of the mature miRNA is incorporated into the RNA-induced silencing complex (RISC), along with Argonaute proteins and the TAR RNA-binding protein (TRBP). This complex identifies target mRNAs by complementary base pairing between the miRNA and sequences typically located in the 3’ untranslated region (3’ UTR) of the mRNA. Binding of RISC to the target mRNA results in either cleavage of the mRNA or inhibition of its translation, thereby preventing protein synthesis (MacFarlane and Murphy, 2010; Michlewski and Cáceres, 2019). 

**Figure 3:**
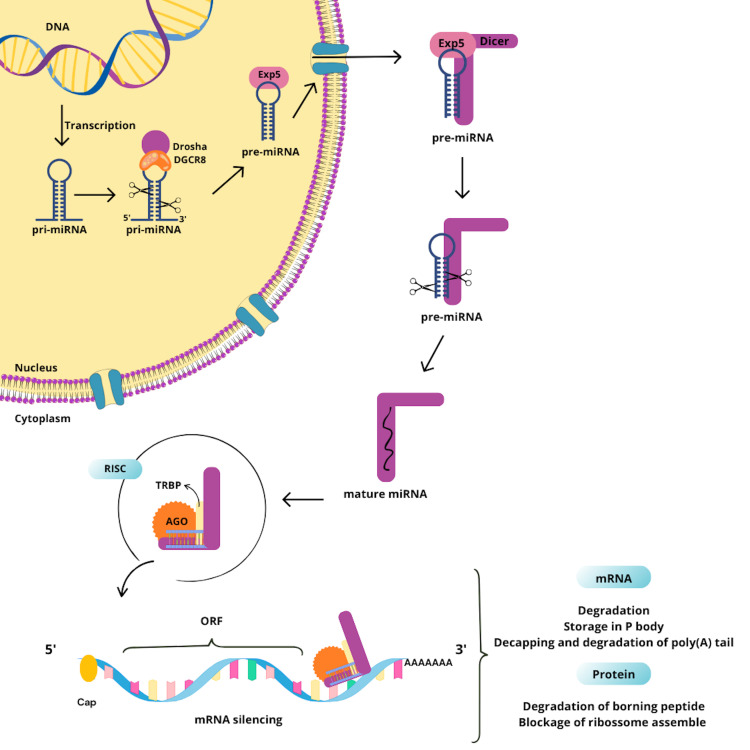
Stages of nuclear and cytoplasmic biogenesis of miRNAs and their role in gene silencing. miRNA processing: From DNA, pri-miRNAs are generated, then pre-miRNAs, which in the cytoplasm are cut by the enzyme Dicer into mature molecules. These are associated with the RISC complex to repress protein synthesis, degrading the mRNA or blocking its translation. DGCR8: DiGeorge Syndrome Critical Region 8; TRBP: TAR RNA Binding Protein; AGO: Argonaute; ORF: Open Reading Frame.

Thus, miRNAs serve as key post-transcriptional regulators of gene expression, playing essential roles in numerous biological processes (MacFarlane and Murphy, 2010; Michlewski and Cáceres, 2019) (Figure 3).

Circular RNAs (circRNAs) are characterized by a covalently closed loop structure, lacking exposed 3′ and 5′ ends. They are generated through a process known as back-splicing and can be classified into four main types based on their genomic origin: exonic circRNAs, intronic circRNAs, exon-intron circRNAs (EIciRNAs), and intergenic circRNAs (Wang et al., 2020; Chen and Shan, 2021). 

**Figure 4:**
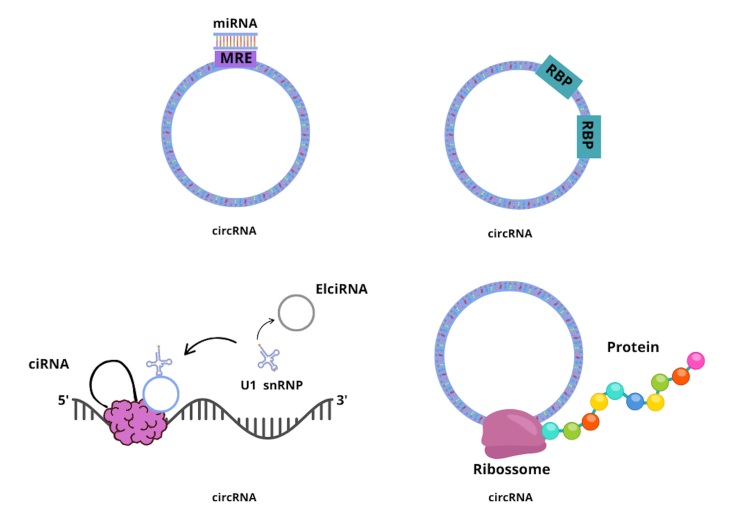
Mechanisms of action and biological functions of circRNAs. Proposed mechanisms of action of circRNAs: acting as miRNA sponges, interaction with RBPs modulating mRNA processing, regulation of transcription and translation into proteins. MRE: miRNA binding sites; RBP: RNA-binding proteins; snRNPs: small nuclear U1 ribonucleoproteins; EIciRNAs: exon-intron circRNAs.

CircRNAs are involved in various pathophysiological processes. One of their key functions is acting as microRNA (miRNA) sponges, as they contain multiple miRNA response elements (MREs) that competitively bind miRNAs and prevent their interaction with target mRNAs at the 3′ untranslated region (3′ UTR). In addition, circRNAs can bind RNA-binding proteins (RBPs), thereby modulating mRNA processing, including splicing and stability (Kumari et al., 2022). Some circRNAs also regulate the transcription of their parental genes. For example, circular intronic RNAs (ciRNAs) can interact with RNA polymerase II (Pol II) to influence transcriptional activity. Similarly, exon-intron circRNAs (EIciRNAs) associate with small nuclear ribonucleoproteins (snRNPs), such as U1, and subsequently with Pol II to modulate transcription. Furthermore, although traditionally considered non-coding, certain circRNAs have been shown to be translated by ribosomes, resulting in the production of functional peptides or proteins (Beermann et al., 2016; Jin et al., 2020; Sun et al., 2020; Qian et al., 2021) (Figure 4).

The regulatory functions of ncRNAs are essential for maintaining cellular homeostasis. Consequently, their dysregulation has been widely implicated in the pathogenesis of various human diseases, including cancer, cardiovascular disorders, neurological conditions, infectious diseases, and metabolic syndromes (Mendell and Olson, 2012; Loganathan and Doss, 2023). 

In this context, investigating the potential interactions between bioactive compounds found in essential oils and ncRNAs represents an emerging and innovative frontier in biotechnology and molecular pharmacology. The modulation of ncRNA expression by essential oil constituents can influence key signaling pathways involved in inflammation, apoptosis, cell proliferation, and immune responses. Exploring these interactions may contribute significantly to the development of more targeted, effective, and less toxic natural therapeutic strategies.

## Methods

To provide a comprehensive and structured overview of the current evidence, this review aimed to summarize how essential oils regulate the expression of long non-coding RNAs (lncRNAs) in human diseases and to highlight the therapeutic implications of these interactions. To this end, we conducted a narrative, non-systematic search in a major scientific database (PubMed), focusing exclusively on studies that investigated essential oils and their ability to modulate lncRNA expression in the context of human physiological or pathological conditions. Articles were included if they (i) evaluated essential oils or their isolated bioactive constituents, (ii) examined their effects on lncRNAs, and (iii) reported outcomes related to human diseases or human-derived models. Additionally, only studies published within the past 15 years were considered eligible. This methodological approach allowed us to integrate and critically discuss the most relevant findings linking essential oils to lncRNA-mediated regulatory mechanisms in human health and disease.

## Modulation of ncRNAs by essential oils in human diseases

### lncRNAs

*Cancer*

*Chemotherapy remains a cornerstone in the treatment of various types of cancer. However, a significant proportion of patients experience tumor recurrence, often associated with drug resistance and/or multi-organ toxicity (* *Ramsey* et al., 2020). In this context, essential oils have garnered increasing attention due to their wide range of bioactive properties, including antioxidant, antimutagenic, antibacterial, anti-inflammatory, antiviral, antidepressant, and anticancer activities (Liu et al., 2019). Consequently, the combination of essential oils with conventional chemotherapy is being increasingly investigated as a strategy to enhance treatment efficacy while potentially mitigating adverse effects (Yuan et al., 2017). 

*Mentha suaveolens × piperita* (grapefruit mint) is a hybrid, perennial aromatic plant extensively cultivated worldwide, primarily for its essential oil, which is widely used in the food, cosmetic, and pharmaceutical industries. Recent studies have demonstrated that its essential oil induces programmed cell death through apoptosis. Moreover, wound healing scratch assays have confirmed its anti-migratory and anti-invasive effects. Notably, the essential oil was found to upregulate the expression of Maternally Expressed Gene 3 (MEG3), a tumor-suppressor lncRNA involved in the regulation of cell proliferation, growth, and metastasis in breast cancer cell lines (Jahanafrooz et al., 2024). 

### miRNA

*Cancer*

*Triple-negative breast cancer (TNBC) accounts for approximately 15% of breast cancer cases and is associated with the poorest prognosis and a high rate of recurrence (* *Gluz* et al., 2009; Foulkes et al., 2010). Patients with TNBC often receive chemotherapy in combination with natural products to overcome chemoresistance (Tang et al., 2016). *Rhizoma curcumae* essential oil, whose main active component is curcumol, has demonstrated the potential to enhance the therapeutic efficacy of doxorubicin chemotherapy in breast cancer cells. This effect is linked to the upregulation of miR-181b-2-3p by curcumol, which directly targets the 3’ UTR of ABCC3 mRNA, resulting in decreased expression of ABCC3, a member of the ATP-binding cassette (ABC) transporter family involved in drug efflux (Zeng et al., 2020). 

Similarly, *Brucea javanica* oil, rich in fat-soluble tetracyclic triterpene quassinoids, has been recognized for its anticancer potential in treating malignant tumors (Cui et al., 2010). In hepatocellular carcinoma models, both *Brucea javanica* oil and its brusatol-enriched formulation induce apoptosis in H22 cells, mediated by upregulation of miRNA-29b, increased expression of p53, Bax, and Bad, and decreased expression of the anti-apoptotic protein Bcl-2. Elevated p53 levels promote Bax expression, which downregulates Bcl-2, leading to mitochondrial outer membrane permeabilization, cytochrome c release, and subsequent activation of caspases-9 and -3. This cascade culminates in PARP cleavage, facilitating apoptosis induction in H22 cells. These molecular effects correlated with a significant extension of survival time in mice bearing H22 ascites tumors treated with *Brucea javanica* oil or its enriched formulation. Nevertheless, further research is warranted to clarify the detailed mechanisms and to investigate other bioactivities of *Brucea javanica* oil in various malignancies (Wang et al., 2021a). 

Building on the antitumor potential of essential oils, *Cinnamomum zeylanicum* essential oil, rich in cinnamaldehyde, eugenol, and other bioactive compounds, significantly reduced tumor volume and mitotic index in murine breast carcinoma models. Treated tumors exhibited increased expression of caspase-3 and Bax, along with reductions in Bcl-2, Ki67, VEGF, and CD24. *In vivo* epigenetic analysis revealed that high-dose treatment reduced histone methylation marks H3K4me3 and H3K9me3, and decreased methylation of promoters of the *Ataxia Telangiectasia Mutated* gene and *Tissue Inhibitor of Metalloproteinases 3* (*TIMP3*). Importantly, miR-21 and miR-155, both associated with tumor progression, were downregulated, underscoring the epigenetic modulatory potential of cinnamon oil in breast cancer (Kubatka et al., 2020). 

Exposure of HeLa cervical cancer cells to *Thymus vulgaris* essential oil resulted in pronounced suppression of *MMP2* expression, a gene implicated in tumor invasion and metastasis. Apoptosis was induced via activation of caspase-3 and caspase-8, indicating caspase-dependent cell death pathways. Additionally, *Thymus vulgaris* essential oil treatment upregulated tumor-suppressive microRNAs miR-16 and miR-34a, which regulate cell cycle progression and apoptosis. These findings highlight the potential of *Thymus vulgaris* essential oil as a chemopreventive and therapeutic agent in cervical cancer (Preljević et al., 2024).

In another study, essential oil from *Monarda didyma* L., extracted from flowering aerial parts, was characterized chemically and evaluated for antioxidant and anti-inflammatory properties. The identified chemotype, *Monarda didyma* ct. carvacrol, was associated with reduced expression of the pro-inflammatory cytokine IL-6 and increased levels of miR-146a, suggesting modulation of the Toll-like receptor 4 (TLR4) signaling pathway (Fraternale et al., 2022). 

*Lung disease*

In acute lung injury, pretreatment with β-patchoulene, an active tricyclic sesquiterpene found in patchouli oil (β-PAE), demonstrated promising effects by upregulating miR-146a expression. This upregulation attenuated lipopolysaccharide-induced activation of nuclear factor kappa B (NF-κB) and reduced the production of pro-inflammatory cytokines, including tumor necrosis factor alpha (TNF-α) and interleukins IL-6 and IL-1β. Additionally, miR-146a reactivated the nuclear factor erythroid 2-related factor 2 (Nrf2)-dependent antioxidant pathway. These findings underscore the therapeutic potential of β-PAE, emphasizing the importance of coordinated modulation of inflammatory and antioxidant pathways, along with miRNA regulation, as promising strategies for managing acute lung injury (Chen et al., 2017). 

*Joint diseases*

During the chondrogenic differentiation of mesenchymal stem cells, the enzyme adenosine deaminase acting on RNA type 2 (ADAR2) was shown to promote editing of pre-miR-181a-5p by converting adenosine to inosine (read as guanosine). This RNA editing alters the function of miR-181a-5p, directing it to inhibit the transcription factor YY1 (Yin Yang 1). Reduced YY1 activity impairs the transcription of *SOX9* (*SRY-Box Transcription Factor 9*), a critical gene marker for chondrocytes that regulates the expression of cartilage-specific genes, resulting in decreased chondrogenic differentiation (Nakamura et al., 2016; Peng et al., 2018). 

The present study demonstrated that essential oil extracted from *Atractylodes lancea* specifically inhibits miR-181a-5p expression and, synergistically, promotes the A→G base substitution in pre-miR-181a-5p associated with ADAR2 activity. Thus, *Atractylodes lancea* essential oil acts upstream by modulating both the expression and editing of miR-181a-5p via ADAR2, neutralizing its inhibitory effect on the YY1-SOX9 pathway and promoting chondrogenic differentiation, highlighting its therapeutic potential in bone diseases (Ye et al., 2023).

*Cognitive function*

Zhao et al. (2024) suggest that the volatile oil of *Angelica sinensis* Radix may modulate cognitive function by downregulating miR-301a-3p, which leads to increased expression of Ppp2ca and other key proteins involved in synaptic plasticity. *Angelica sinensis* Radix, a widely used herb in traditional Chinese medicine, is traditionally valued for its blood-tonifying properties, its ability to promote circulation, and its effectiveness in alleviating pain related to menstrual disorders (Zhao *et al*., 2024). 

### circRNA

Despite the growing interest in non-coding RNAs as key regulatory elements in human diseases, our literature search revealed a notable scarcity of studies investigating the effects of essential oils on circRNAs. Although circRNAs are recognized for their roles in transcriptional regulation, microRNA sponging, and modulation of protein interactions, we found no substantial experimental evidence linking essential oils to circRNA expression or function in human-derived models (Kumari et al., 2022). This paucity of data prevents a more detailed or integrative discussion similar to that provided for miRNAs and lncRNAs. Therefore, circRNAs currently represent an underexplored regulatory layer in the context of essential oils and human diseases, highlighting a significant gap that warrants future experimental investigation.

## Future Perspectives

Essential oils have emerged as promising bioactive compounds in pharmaceutical research, especially with advances in nanoencapsulation technologies that enable enhanced stability, controlled release, and improved bioavailability. Due to their high volatility and chemical instability, essential oils are prone to degradation when exposed to light, oxygen, moisture, and temperature fluctuations. Nanoencapsulation offers a sophisticated strategy to overcome these limitations by protecting the biochemical integrity of essential oils, preserving their pharmacological properties, and facilitating their safer and more effective therapeutic use (Arora et al., 2025). 

Concurrently, essential oils have been linked to the modulation of ncRNAs, such as miRNAs and lncRNAs, which play central roles in the post-transcriptional regulation of gene expression and the pathophysiology of various diseases. The interactions between miRNAs and lncRNAs form a critical epigenetic regulatory network capable of influencing diverse cellular processes. Thus, identifying and characterizing miRNA-lncRNA interactions modulated by essential oils is crucial for elucidating their mechanisms of action and therapeutic potential (Liu et al., 2025). 

Within this context, a promising yet underexplored area is the role of essential oils in modulating ncRNAs during wound healing. Evidence shows that *lavender* essential oil activates the TGF-β pathway, promoting collagen synthesis, fibroblast differentiation, and tissue contraction (Mori et al., 2016), while *Cinnamomum verum* (cinnamon) oil enhances cell proliferation, re-epithelialization, and inflammation reduction via activation of growth factors such as IGF-1, FGF-2, and VEGF (Seyed Ahmadi et al., 2019). Despite these beneficial effects during the early healing stages, the impact of essential oils on complete re-epithelialization and long-term tissue remodeling remains uncertain. Given that miRNAs, lncRNAs, and circRNAs directly regulate pathways involved in these processes, including TGF-β signaling, future research exploring essential oils’ capacity to modulate these ncRNAs may uncover novel molecular mechanisms and guide the development of nanoencapsulated essential oil-based adjuvants in tissue regeneration therapies (Essandoh et al., 2016; Mori *et al*., 2016; Seyed Ahmadi *et al*., 2019). 

Although no studies have been identified that investigate the effects of nanoencapsulated essential oils on ncRNA regulation, current evidence demonstrates that nanotechnological strategies can substantially enhance the physicochemical and biological properties of essential oils. Encapsulation has been shown to improve thermal and chemical stability, increase bioavailability, and enable the controlled release of volatile constituents. For example, nanostructured systems have facilitated the controlled release of compounds from *Achyrocline satureioides* essential oil (da Silva et al., 2024), and thermogravimetric analyses have shown that encapsulated *Ferulago angulata* (chavir) essential oil decomposes at higher temperatures than the free oil, confirming successful stabilization provided by the nanocapsules (Kaboudi et al., 2023). These findings strengthen the rationale for proposing nanoencapsulation as a promising technological approach to enhance the therapeutic potential of essential oils, even though its effects on ncRNA modulation remain an unexplored research frontier.

## Conclusion

**Figure 5:**
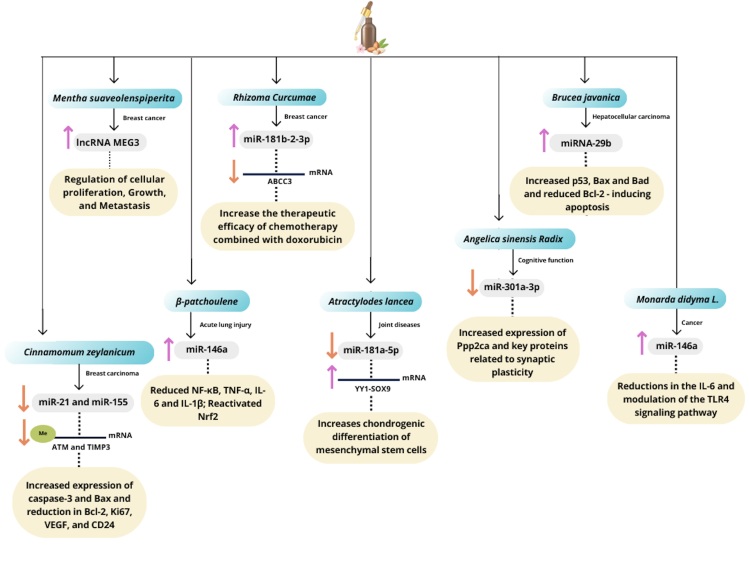
Molecular effects of essential oils on lncRNAs and miRNAs. Schematic representation of the main molecular mechanisms by which essential oils modulate the expression and function of lncRNAs and miRNAs, influencing signaling pathways and associated cellular processes. MEG3: Maternally Expressed Gene 3; ABCC3: ATP Binding Cassette Subfamily C Member 3; Me: Methylation; ATM: Ataxia Telangiectasia Mutated; TIMP3: Tissue Inhibitor of Metalloproteinases 3; YY1: Yin Yang 1 transcription factor; SOX9: SRY-Box Transcription Factor 9.

The use of essential oils to modulate ncRNAs represents a promising and rapidly evolving field in pharmacological research, with potential applications across a wide range of diseases, including cancer, acute lung injury, joint disorders, and neurocognitive conditions. Technological advances, such as nanoencapsulation, offer solutions to the inherent challenges of essential oil volatility and chemical instability, thereby enhancing their therapeutic efficacy and safety. The ability of essential oils to regulate miRNAs, lncRNAs, and potentially circRNAs opens new avenues for the development of innovative therapeutic strategies targeting complex biological processes such as apoptosis, drug resistance, inflammation, cell differentiation, and synaptic plasticity. Figure 5 provides a detailed compilation of the main findings, integrating the molecular mechanisms and biological effects reported across the studies analyzed. Nevertheless, significant gaps remain, particularly regarding the interactions between essential oils and circRNAs, as well as the clinical translation of these findings. Further experimental and clinical research is essential to advance our understanding of these epigenetic mechanisms and to support the integration of essential oils as therapeutic adjuvants in modern medicine.

## Data Availability

Data availability is not applicable to this article as no new data were created or analyzed in this study.
